# Konjac glucomannan polysaccharide and inulin oligosaccharide ameliorate dextran sodium sulfate-induced colitis and alterations in fecal microbiota and short-chain fatty acids in C57BL/6J mice

**DOI:** 10.37796/2211-8039.1191

**Published:** 2021-09-01

**Authors:** Chih-Hsuan Changchien, Cheng-Hsin Wang, Hsiao-Ling Chen

**Affiliations:** 1 Department of Medical Laboratory Science and Biotechnology, Asia University, Taiwan; 2 Department of Plastic and Reconstructive Surgery, Ditmanson Medical Foundation Chia-Yi Christian Hospital, Taiwan; 3 Department of Food and Nutrition, Providence University, Taiwan; 4 Department of Food, Nutrition and Health Biotechnology, Asia University, Taiwan; 5 Department of Medical Research, China Medical University Hospital, China Medical University, Taiwan

**Keywords:** Colitis, Dextran sulfate sodium, Konjac glucomannan, Inulin, Microbiota, Mucosal barrier

## Abstract

**Background:**

Konjac glucomannan polysaccharide (KGM), inulin oligosaccharide (inulin) and their mixture has been shown to modulate the gut-associated lymphoid tissue immunity.

**Aims:**

The present study was mainly to determine effects of a low-level (2% w/w) KGM and inulin and their combination on dextran sodium sulfate (DSS)-induced colitis. We also determine the potential mechanisms mediating these effects of dietary fibers.

**Methods:**

C57BL/6J mice (6 weeks of age, eight per group) were randomly assigned to consume one of the following diets: control (DSS group) or control diet supplemented with 2% (w/w) of KGM (KGM group), 2% (w/w) of inulin oligosaccharide (inulin group) or KGM+Inulin (1%, w/w each (K+I group)) for 29 days, combined with the DSS drinking water (2% w/v) treatment on days 21–26. Another group served as vehicle was fed the control diet and given regular drinking water throughout the study. Fresh feces were collected on days 26–29. Mice were killed on day 30 after fasting. Segments of distal colon were processed for histological procedure. The remaining colonic tissues were processed to determine the colonic gene expressions of cytokines, tight junction proteins and antioxidant enzymes.

**Results:**

The present study indicated that DSS resulted in colonic dysplasia, severe leukocyte infiltration and enhanced gene expressions of pro-inflammatory cytokines. All fiber treatments ameliorated these indices of colitis. DSS treatment reduced the colonic gene expressions of tight junction proteins and antioxidant enzymes, which were ameliorated or normalized with fiber supplementation. In addition, all fiber treatments prevented the DSS-induced alterations in the fecal microbiota and short-chain acid levels.

**Conclusion:**

Supplementation of low-level, 2% (w/w), of KGM polysaccharide, inulin oligosaccharide and K + I reduced the DSS-induced colitis and mucosal barrier dysfunction, which was likely to be mediated by the prebiotic effects.

## Introduction

The ulcerative colitis is an inflammatory bowel disease (IBD) that occurs in the colon [1]. Epidemiological studies found that the incidence and prevalence of IBD were the highest in western countries [2]. The reported annual incidence of ulcerative colitis was 24.3 and 19.2 per 100,000 person-years in Europe and North America, respectively [2]. The incidence is still arising in low-incidence areas such as Asia and developing countries [2]. The pathogenesis of IBD is multifactorial, involving mucosal barrier defects, dysregulated immune responses, genetic predisposition and environmental factors [3].

The mucosal barrier consists of mucosal layer, tight junction and microflora, which protects the body from potential pathogens [4]. The tight junctions form the continuous intercellular barrier between the intestinal epithelial cells is required to separate tissue spaces, regulate selective movement of solutes across the epithelium and maintain the mucosal barrier function [4]. The reduced expression of tight junction protein may lead to translocation of endotoxins and bacterial antigens and causes excessive inflammation in the gut-associated lymphocyte tissue [4].

The colonic mucosa is constantly challenged by the reactive oxidative substances produced from the microbiota and lumen contents [5]. An imbalanced cellular redox system of mucosa results excess reactive oxidative substance levels and subsequently causes gastrointestinal tract inflammation [5,6]. In addition, activated leukocytes during the active phase of inflammation generate reactive oxidative reactions that further impairs the mucosal barrier [7]. Therefore, oxidative stress is considered to play an important role in the mucosal damage in IBD [5]. For examples, previous studies indicated increased peroxidative products in both the ulcerative colitis mouse model and IBD patients [8,9].

Current medical treatments for IBD include corticosteroids, antibiotics, 5-aminosalicylic acid, immunomodulator and biological therapies [10]. Traditional therapy may cause side effects due to immune-suppression [11]. Therefore, the need for complementary and alternative medicine approaches for prevention and treatment of IBDis increasing [12].

Konjac glucomannan (KGM) is a viscous soluble fiber isolated from the underground tuber of *Amorphophallus konjac* [13]. It is composed of *β*-1, 4-linked D-glucose and D-mannose units joined together with branches through *β*-1,6-glucosyl units [13]. The KGM has been processed into various vegetarian food products commonly consumed in the Asian countries. Inulin, found in the underground tuber of chicory (*Cichorium intybus*), is an oligomer or polymer of *β*-D-fructose containing two to mostly sixty monomeric units that are linked by *β*-(2 → 1) glycosidic bridges [14]. The fraction of inulin with the degree of polymerization lower than 10, namely, inulin oligosaccharide, is widely used as a supplement in functional food [15]. Both KGM and inulin oligosaccharide have been shown to exert healthy bowel effects, such as to increase the colonic microbial fermentation products short-chain fatty acids and stimulate the growth of bifidobacteria and lactobacilli in animal/human studies [14,16–18]. KGM and inulin modulate the colonic antioxidant system and increased the antioxidant defense against the oxidative stress resulted from a low-fiber high-fat diet or a genotoxic carcinogen [18–20]. In addition, a previous study indicated that either KGM or inulin oligosaccharide could effectively strengthen the colonic mucosal barrier and increase the expressions of tight junction proteins occludin (a transmembrane barrier protein) and zone occludin-1 (ZO-1) (a peripheral scaffolding protein) [18]. These fibers also modulate gut immunity by increasing the colonic gene expression of IL-10, an anti-inflammatory cytokine [16]. However, effects of KGM and the combination of KGM and inulin oligosaccharide on prevention of ulcerative colitis have never been examined.

Dextran sodium sulfate (DSS) causes colonic intestinal epithelial cell injury and has been used to induce experimental colitis [21,22]. The present study was undertaken to investigate effects of KGM, inulin oligosaccharide and their mixture on the colonic inflammation, tight junction proteins, antioxidative enzymes, fecal microbiota and short-chain fatty acids in a DSS-induced colitis mouse model. In order to be applicable to humans, the level of fiber supplementation was only 2% w/w, which is equivalent to 10 g/d of normal human diet.

## Methods

### Animals

Male C57BL/6J mice were purchased at 5 weeks of age from the National Laboratory Animal Breeding and Research Center (Taipei, Taiwan). Animals were housed in a solid-bottomed plastic cage with stainless wire bar lid and wood shavings for bedding in an animal holding room maintained on a 12-h light/dark cycle at 24 ± 1°C and 50 ± 2% humidity. All animals were allowed free access to water and food in the present study. The care and use of animals was in accordance with European Commission Directive 86/609/EEC for animal experiment [23] and the guidelines of the National Research Council [24]. All experimental procedures involving animals were approved by the Institutional Animal Care and Use Committee.

### Experimental design

Mice (6 weeks of age, *n* = 8 per group) were randomly assigned into one of the following groups: vehicle, DSS, KGM, inulin and K+I. The vehicle group consumed the control diet and regular drinking water. The other mice were DSS-treated groups; they were fed one of following diets: control (DSS group) or fiber-supplemented diet (2% w/w KGM (KGM group), 2% w/w inulin oligosaccharide (inulin group), 1% w/w KGM+1% w/w inulin oligosaccharide (K+I group)) for 29 days and were given 2% (w/v) DSS (molecular weight 36,000–50,000, MP Biomedicals, Santa Ana, CA, USA) in drinking water on days 21–26. The control diet was modified from AIN-93G [25]. Compositions of all experimental diets are shown in Table 1. The fiber-supplemented diets were adjusted based on the purity of konjac and inulin powder to provide 2% (w/w) dietary fiber supplementation. On days 26–29, mice were individually housed for collection of fresh feces. Mice were anaesthetized with CO_2_ and killed on day 30 after an 18-h fasting. Segments (0.4 cm^2^) of the distal colon were immediately fixed in 10% (v/v) buffered formalin for histological procedure. The remaining tissues were frozen for further analysis.

### Histological evaluation of distal colon

Tissue sections (4 μm) were stained with hematoxylin and eosin. The histology of colonic mucosal layer was observed under a 200 × magnification.

### Determination of gene expression

The colonic gene expressions of cytokines (TNF-α, IL-6 and IL-10), tight junction proteins (ZO-1 and occludin) and antioxidant enzymes including glutathione peroxidase 2 (Gpx2), glutathione *S*-transferase π (Gstp1), catalase and superoxide dismutase (SOD) were determined using TaqMan gene expression assays (Applied Biosystems Life Technologies, Foster City, CA, USA) by the method described previously [18]. The TaqMan primer identification numbers of genes were provided in the supplementary material. The quantitative real-time polymerase chain reaction was performed at 50°C for 2 min, 95°C for 10 min, and fifty cycles at 95°C for 15 s and 60°C for 1 min. The relative gene expression of each target gene was first normalized to that of glyceraldehyde 3-phosphate dehydrogenase (GAPDH) and further calculated relative to the level of the vehicle group using the comparative threshold cycle (C_t_) method. The fold difference in gene expression was calculated as 2^−ΔΔ^*^C^*^t^ [26].

### Determination of fecal short-chain fatty acids

Fecal acetate, propionate and *n*-butyrate were extracted using ether by the method described previously [18]. The fecal samples were injected onto a gas chromatography (GC-2014, Shimadzu, Tokyo, Japan) fitted with a glass capillary column (0.25 mm, 30 m Stabilwax-DA, Restek Corp., Bellefonte, PA, USA) and a flame ionization detector by the method described previously [18].

### Quantification of fecal bacteria

Fecal bifidobacteria, lactobacilli and Clostridia were quantified by the fluorescent *in situ* hybridization method as described previously [18, 27–28]. A nucleic acid stain, 4′, 6-diamidino-2-phenylindole was used to quantify the total fecal bacteria. The microbial counts are expressed as log_10_ counts/g feces.

### Statistical Analyses

Data were presented as means and standard error of means (S.E.M.) and analyzed using SPSS version 12. The body weights were analyzed using two-way ANOVA with day and treatment factors. One-way ANOVA followed by *post hoc* Tukey’s honestly significant difference test was used to determine variables across groups. Statistically significant was defined as *P* < 0.05.

## Results

**Figure 1:**
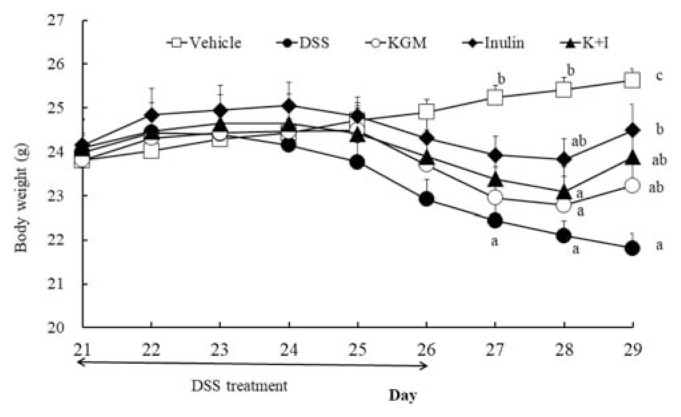
Body weight of vehicle and DSS-treated experimental mice during days 21–29. Mean values with different superscript letter indicated significantly different across groups (P < 0.05).

The body weights from the initial day of the DSS treatment (day 21) to the end of the study is shown in Fig. 1. Two-way ANOVA indicated significant effect of day (*P* < 0.001) and interaction between day and treatment (*P* = 0.045). Further post-hoc statistical analysis across groups indicated that the body weight of the DSS group was lower than the respective level of the vehicle group on day 27 (*P* = 0.004), day 28 (*P* = 0.001) and day 29 (*P* < 0.001), respectively. The body weight of inulin group was greater (*P* = 0.004 vs DSS) than the level of DSS group on day 29. KGM and K+I did not significantly affect the body weight compared with the respective level of DSS group during days 21–29.

**Figure 2:**
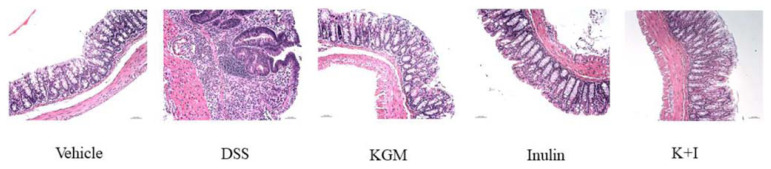
Histology of the distal colon of vehicle and DSS-induced mice. Histology of the distal colon was observed under a 200× magnification. Scale represents 50 μm.

The cross-section histology of distal colon indicated severe dysplasia in the DSS group, which was not observed in the vehicle and fiber-supplemented groups (Fig. 2). In addition, the present study found severe leukocyte infiltration throughout the lamina propria and extending into the submucosal tissues in the DSS group, which was not observed in the vehicle and was ameliorated in fiber-supplemented groups.

**Figure 3:**
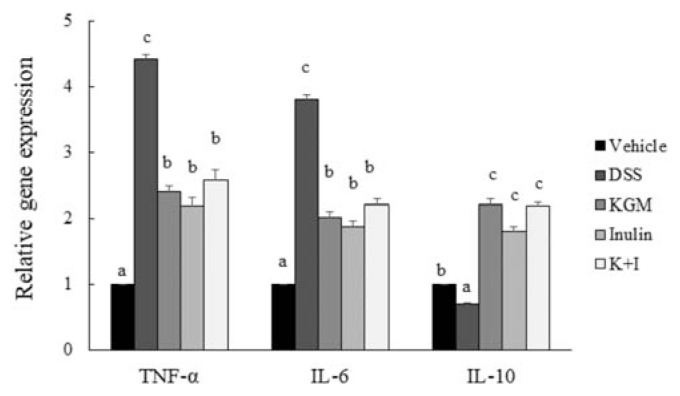
Gene expressions of cytokines in the distal colon of vehicle and DSS-induced mice. Relative gene expression was normalized using internal housekeeping gene GAPDH and compared to that of the vehicle group according to the 2^−ΔΔCt^ method. Bars are presented as mean and standard error of means (n 8 per group). Mean values with unlike letters are significantly different across groups as analyzed by one-way ANOVA followed by Tukey’s honestly significant difference test (P < 0.05).

The DSS treatment significantly up-regulated the colonic gene expression of TNF-α (*P* < 0.001 vs vehicle) and IL-6 (*P* < 0.001 vs vehicle), respectively (Fig. 3). The gene expression of TNF-α in the KGM, inulin or K+I was significantly lower than that in the DSS group (*P* < 0.001 for each fiber vs DSS, respectively). Similarly, the gene expressions of IL-6 in all fiber-supplemented groups were significantly lower than that in the DSS group (*P* < 0.001 for each fiber vs DSS, respectively). On the other hand, DSS treatment significantly reduced the colonic gene expression of IL-10 (*P* < 0.001 vs vehicle). The gene expression of IL-10 in the KGM, inulin and K+I group was significantly greater than the level in the DSS group, respectively (*P* < 0.001 for each fiber vs DSS, respectively).

**Figure 4:**
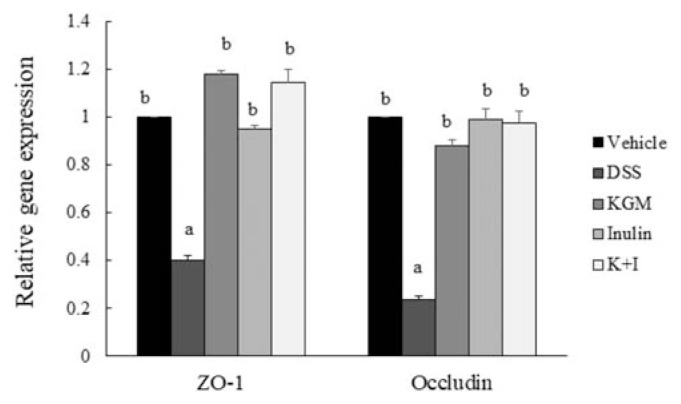
Gene expressions of tight junction proteins in the distal colon of vehicle and DSS-induced mice. Relative gene expression was normalized using internal control gene glyceraldehyde 3-phosphate dehydrogenase (GAPDH) and compared with that of the vehicle group according to the 2^−ΔΔCt^ method. Bars are presented as mean and standard error of means (n 8 per group). Mean values with unlike letters are significantly different across groups as analyzed by one-way ANOVA followed by Tukey’s honestly significant difference test (P < 0.05).

The DSS treatment significantly down-regulated the gene expressions of tight junction proteins ZO-1 (*P* < 0.001 vs vehicle) and occludin (*P* < 0.001 vs vehicle) (Fig. 4). Supplementation of KGM, inulin and K+I up-regulated the *ZO-1* gene expression, respectively (*P* < 0.001 for each fiber vs DSS, respectively). All fiber supplementations also enhanced the *occludin* gene expressions (*P* < 0.001 for each fiber vs DSS, respectively).

**Figure 5:**
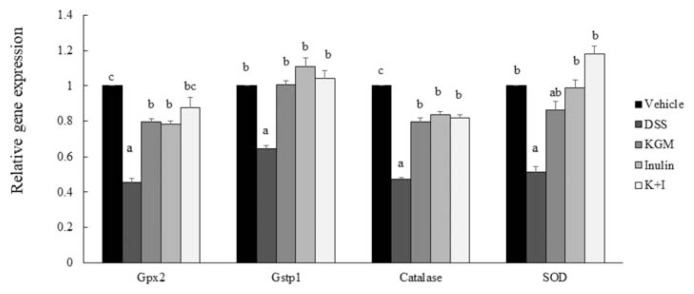
Gene expressions of glutathione peroxidase (Gpx2), glutathione S-transferase π (Gstp1), catalase and superoxide dismutase (SOD) in the distal colon of vehicle and DSS-induced mice. Relative gene expression was normalized using internal control gene glyceraldehyde 3-phosphate dehydrogenase (GAPDH) and compared to that of the vehicle group according to the 2^−ΔΔCt^ method. Bars are presented as mean and standard error of means (n 8 per group). Mean values with unlike letters are significantly different across groups as analyzed by one-way ANOVA followed by Tukey’s honestly significant difference test (P < 0.05).

The DSS treatment significantly down-regulated the colonic gene expression of glutathione peroxidase (*P* < 0.001 vs vehicle), glutathione *S*-transferase (*P* < 0.001 vs vehicle), catalase (*P* < 0.001 vs vehicle) and SOD (*P* < 0.001 vs vehicle), respectively (Fig. 5). KGM, inulin and K+I ameliorated the DSS-induced suppression of glutathione peroxidase gene expression (*P* < 0.001 vs DSS, respectively) and catalase gene expression (*P* < 0.001 vs DSS, respectively). The gene expressions of glutathione *S-*transferase in all fiber groups were normalized to the level in the vehicle group (*P* > 0.05 for each fiber vs vehicle, respectively), respectively. The gene expressions of SOD in all fiber groups were also similar to that in the vehicle group (*P* > 0.05 for each fiber vs vehicle, respectively).

**Figure 6:**
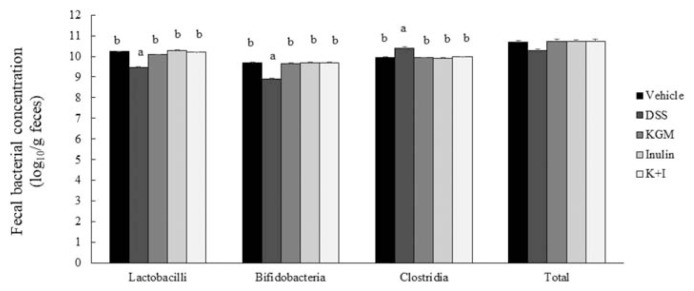
Fecal bacterial concentrations (log_10_ counts/g feces). Bars are presented as mean and standard error of means (n 8 per group). Mean values with unlike letters are significantly different across groups as analyzed by one-way ANOVA followed by Tukey’s honestly significant difference test (P < 0.05). The fecal total bacterial concentrations were not significantly different across groups.

The DSS treatment decreased the fecal lactobacilli (*P* < 0.001 vs vehicle) and bifidobacteria (*P* < 0.001 vs vehicle) concentrations (log_10_ counts/g feces) and increased the Clostridia concentration (*P* < 0.001 vs vehicle) compared with the respective level in the vehicle group (Fig. 6). The fecal microbiota was normalized with all fiber treatments. However, the fecal total bacterial concentration was not affected with the DSS treatment and was similar across groups.

The DSS treatment decreased the fecal acetate, propionate, butyrate and total short-chain fatty acid concentrations compared with the respective level in the vehicle group (*P* < 0.001 vs vehicle, respectively) (Table 2). KGM, inulin and K+I increased the fecal acetate by approximately 66.0% (*P* = 0.012 vs DSS), 47.0% (*P* = 0.035 vs DSS) and 59.1% (*P* = 0.028 vs DSS), respectively, compared with the level in the DSS group. KGM, inulin and K+I increased the fecal propionate by approximately by 54.2% (*P* = 0.030 vs DSS), 75.0% (*P* < 0.001 vs DSS) and 64.6% (*P* = 0.010 vs DSS), respectively, compared with the level in the DSS group. KGM, inulin and K+I increased the fecal butyrate approximately by 1.3-fold (*P* < 0.001 vs DSS), 2.3-fold (*P* < 0.001 vs DSS) and 2-fold (*P* < 0.001 vs DSS), respectively, compared with the level in the DSS group. KGM, inulin and K+I increased the fecal total short-chain fatty acid by approximately 66.8% (*P* = 0.015 vs DSS), 57.9% (*P* = 0.035 vs DSS) and 65.5% (*P* = 0.018 vs DSS), respectively, compared with the level in the DSS group.

## Discussion

There are several unique aspects in the present study. Firstly, the DSS administered in the present study, 2% w/v, was lower than those used in previous studies, 3–8% w/v in drinking water [29–32]. The DSS-treated period in the present study was also shorter than that shown in previous studies [30,32]. Secondly, the present study determined the preventive effects of dietary fiber on colitis, instead of being an adjunct treatment during the active phase of colitis [29–32]. Thirdly, the present study compared effects of combination of polysaccharide (KGM) and inulin oligosaccharide with single fiber source on DSS-induced colitis. We further examined the potential underlying mechanisms that mediated the anti-colitis effects of these dietary fibers.

In order to set up an optimally low DSS dose for inducing experimental colitis, we examined the outcomes of 2% (w/v) DSS water for 5 days in a pilot study. With this low-dose DSS treatment, we observed severe crypt destruction and leukocyte infiltration in the distal colon, in agreement with the those observed by other studies using higher dose of DSS [29,30]. The present study further indicated that this low-dose DSS treatment caused large increases in the colonic expression of pro-inflammatory cytokines TNF-α and IL-6, in agreement with those observed in previous studies using higher dose of DSS [29–31]. Therefore, the present study suggested that 2% (w/v) DSS water for 5 days was sufficient to induce indices of colonic colitis such as decreased body weight, severe crypt destruction and inflammation in C57BL/6J mice. The colitis was not confirmed by the usual standards, such as the disease activity index, colon length, and occult/ obvious fecal blood in the present study.

Fructo-oligosaccharide and inulin administrations during the active colitis phase induced by DSS have been shown to reduce the colonic mucosal lesion [32–33]. In addition, fructo-oligosaccharide has been shown to exert intestinal anti-inflammatory effect in CD4+CD62L+T cell transfer model of colitis in mice [34]. Our present results indicated that inulin oligosaccharide effectively ameliorate DSS-induced colitis, as evidenced by a normal mucosal lining, a lesser body weight loss, lower expressions of pro-inflammatory cytokines and a higher expression of IL-10 compared with the respective level in the DSS group. Although KGM and K+I did not effectively ameliorate the body weight loss, they prevented mucosal lining damage and effectively modulated colonic immunity. Therefore, the present study suggested that these soluble fibers could ameliorate the pathogenesis of DSS-induced colitis.

The mucosal barrier contains microbiota that could modulate the innate and adaptive immunity of gut-associated lymphoid tissue [35,36]. Beneficial bacteria such as *Bifidobacteria* spp. and *Lactobacillus* spp. is associated with a healthy intestinal homeostasis [37]. In response to these beneficial bacteria, dendritic cells within the lamina propria release TGF-β which activates T regulatory cells to release IL-10 and TGF-β, leading to a more tolerant immune phenotype [37]. The gut microbiota of IBD patients is characterized by a reduced abundance of *Bifidobacteria* spp. and *Lactobacillus* spp. and a higher abundance of *Clostridium difficile* [37]. In agreement with that, the present study observed higher fecal Clostridia and lower lactobacilli and bifidobacteria levels in the DSS group. Several studies have suggested beneficial effects of probiotics, such as VSL#3 (mixture of several probiotics) and a single strain of *Bifidobacterium* spp. on DSS-induced colitis [31,38]. Administration of VSL#3 during the DSS treatment reduced severity of colitis and pro-inflammatory cytokine levels [31]. Administration of *Bifidobacterium* spp. before and during the DSS treatment ameliorated the severity of colitis and reduced the colonic pro-inflammatory cytokine IL-1β [38]. The present results indicated that all fiber treatments prevented the DSS-induced alteration in the fecal microbiota. Therefore, the anti-colitis effects of soluble fiber treatments in the present study may be mediated by their prebiotic roles [39].

Inflammatory bowel disease is likely to be triggered by a leaky mucosal layer [4]. In this study, the DSS treatment down-regulated the gene expressions of tight junction proteins, which suggested that DSS might damage the mucosal barrier function. Soluble fibers have been shown to up-regulate the ZO-1 and occludin expressions in the wild-type [18] and the occludin in the CD4+CD62L+T cell transfer model of colitis mice [34]. In accordance with previous studies, the present results indicated that KGM and inulin oligosaccharide could beneficially prevent the DSS-induced down-regulation of ZO-1 and occludin expression, which possibly contribute to their anti-colitis effects.

Previous *in vitro* studies have suggested that the butyrate is vital for normal colonocyte proliferation and increases the transcription of tight junction proteins and induces ZO-1 and occludin redistributions in the cellular membrane [40–41]. In the present study, all fiber treatments significantly enhanced the fecal butyrate level. Therefore, we suggested that the butyrate derived from the dietary fibers might prevent the DSS-induced colonic mucosal dysplasia and the mucosal layer damage.

The present study indicated reduced expressions of colonic antioxidant enzymes in the DSS group, which suggested an impaired colonic antioxidant defense system in this colitis model. We have previously reported that 2% (w/w) KGM, inulin oligosaccharide and K+I up-regulated the expressions of colonic antioxidant enzymes [18]. The present study further indicated that these dietary fibers did not just ameliorate the DSS-induced down-regulation in colonic antioxidant enzymes, but also almost normalized the expressions of glutathione *S*-transferase and SOD to the level of the vehicle group. Therefore, the present study suggested that KGM, inulin oligosaccharide and K+I pretreatment strengthened the colonic antioxidant defense system against the challenge caused by DSS.

In conclusion, the present study suggests that a low-level, 2% (w/w), of KGM polysaccharide, inulin oligosaccharide and the fiber mixture may prevent the DSS-induced colitis, which is likely to be associated with the prebiotic role of these fibers.

## Figures and Tables

**Table 1:** Composition of experimental diets.

Group	Control^1^ (g/kg)	KGM (g/kg)	Inulin (g/kg)	K + I (g/kg)
Maize starch	529.5	504.5	506.1	505.3
Casein	200.0	200.0	200.0	200.0
Sucrose	100.0	100.0	100.0	100.0
Soybean oil	70.0	70.0	70.0	70.0
Cellulose	50.0	50.0	50.0	50.0
Inulin oligosaccharide	-	-	23.4	11.7
Konjac glucomannan	-	25.0	-	12.5
AIN 93G Mineral Mix	35.0	35.0	35.0	35.0
AIN 93 Vitamin Mix	10.0	10.0	10.0	10.0
L-cystine	3.0	3.0	3.0	3.0
Choline bitartrate	2.5	2.5	2.5	2.5

KGM, Konjac glucomannan; K+I, Konjac glucomannan + inulin oligosaccharide; AIN, American institute of Nutrition.

1 The control diet was modified from AIN-93 G diet [25].

**Table 2:** Fecal short-chain fatty acid concentrations of vehicle and DSS-induced mice.^1^

	Vehicle	DSS	KGM	Inulin	K+I

	μmol/g feces				
Acetate	65.3 ± 0.8^c^	30.6 ± 0.6^a^	50.8 ± 0.8^b^	45.0 ± 0.7^b^	48.7 ± 1.0^b^
Propionate	10.3 ± 0.2^c^	4.8 ± 0.1^a^	7.4 ± 0.4^b^	8.4 ± 0.3^b^	7.9 ± 0.3^b^
Butyrate	3.3 ± 0.2^b^	1.4 ± 0.1^a^	3.2 ± 0.1^b^	4.6 ± 0.1^c^	4.2 ± 0.1^c^
Total	78.9 ± 1.3^c^	36.8 ± 0.8^a^	61.4 ± 1.3^b^	58.1 ± 1.2^b^	60.9 ± 1.4^b^

KGM, Konjac glucomannan; K+I, Konjac glucomannan + inulin oligosaccharide.

1 Data were expressed as mean values and standard error of means (*n 8* per group). The differences across groups were determined using one-way ANOVA followed by *post hoc* Tukey’s honestly significant difference test. Mean values with unlike letters are significantly different across groups as analyzed by one-way ANOVA followed by Tukey’s honestly significant difference test (*P* < 0.05).
